# Pregnancy outcomes of women with previous caesarean sections: Secondary analysis of World Health Organization Multicountry Survey on Maternal and Newborn Health

**DOI:** 10.1038/s41598-019-46153-4

**Published:** 2019-07-05

**Authors:** Chumnan Kietpeerakool, Pisake Lumbiganon, Malinee Laopaiboon, Siwanon Rattanakanokchai, Joshua P Vogel, A Metin Gülmezoglu

**Affiliations:** 1 Department of Obstetrics and Gynaecology, Faculty of Medicine, Khon Kaen University, Khon Kaen, 40002 Thailand; 2 Department of Epidemiology and Biostatistics, Faculty of Public Health, Khon Kaen University, Khon Kaen, 40002 Thailand; 3 UNDP/UNFPA/UNICEF/WHO/World Bank Special Programme of Research, Development and Research Training in Human Reproduction (HRP), Department of Reproductive Health and Research, World Health Organization, Geneva, Switzerland

**Keywords:** Epidemiology, Outcomes research

## Abstract

Secondary analysis of World Health Organization Multicountry Survey on Maternal and Newborn Health (WHOMCS) was undertaken among 173,124 multiparous women to assess the association between previous caesarean sections (CS) and pregnancy outcomes. Maternal outcomes included maternal near miss (MNM), maternal death (MD), severe maternal outcomes (SMO), abnormal placentation, and uterine rupture. Neonatal outcomes were stillbirth, early neonatal death, perinatal death, neonatal near miss (NNM), neonatal intensive care unit (NICU) admission, and preterm birth. Previous CS was associated with increased risks of uterine rupture (adjusted Odds Ratio (aOR); 7.74; 95% confidence interval (CI) 5.48, 10.92); morbidly adherent placenta (aOR 2.60; 95% CI 1.98, 3.40), MNM (aOR 1.91; 95% CI 1.59, 2.28), SMO (aOR 1.80; 95% CI 1.52, 2.13), placenta previa (aOR 1.76; 95% CI 1.49, 2.07). For neonatal outcomes, previous CS was associated with increased risks of NICU admission (aOR 1.31; 95% CI 1.23, 1.39), neonatal near miss (aOR 1.19; 95% CI 1.12, 1.26), preterm birth (aOR 1.07; 95% CI 1.01, 1.14), and decreased risk of macerated stillbirth (aOR 0.80; 95% CI 0.67, 0.95). Previous CS was associated with serious morbidity in future pregnancies. However, these findings should be cautiously interpreted due to lacking data on indications of previous CS.

## Introduction

Caesarean section (CS) is among the essential comprehensive intrapartum services. CS can be a life-saving intervention for the foetus, the mother, or both in certain circumstances including obstructed labour, distressed foetus, obstetric haemorrhage, abnormal presentation, and other emergency obstetric conditions. An appropriate provision of CS can avert either maternal or neonatal deaths^1,2^. In the countries with a low country-level of CS rates, increasing CS could preclude approximately 160,000 maternal deaths and 800,000 neonatal deaths per annum^1^. In addition, 60% of the maternal mortality among pregnant women in low-income countries could be prevented if CS was performed at a population level of 10–15%^2^.

Based on the World Health Organization (WHO) global survey, the CS rates varied widely across the geographical regions, with country-level rates ranging from less than 10% to more than 50%^3–5^. The CS rates were lowest among the African countries with a median rate of 8.8% indicating the limited use of CS in the African health facilities surveyed^3^. The median rate of CS among the countries in Latin America was 33%, with the highest rates reported in private hospital settings (51%). The high CS rates in private institutions in Latin America were mostly because of an increase in elective CS^4^. Data obtained from nine countries in Asia noted a 27.3% overall CS rate among 122 recruited facilities^5^. The highest CS rate was in China (46.2%) followed by Vietnam (35.6%), and Thailand (34.1%). In addition, the rate of CS performed without a medical indication was highest in China (11.7%) followed by Vietnam (1.0%), and Sri Lanka (0.8%)^5^. When medically indicated, a CS can reduce serious risks of maternal and perinatal mortality and morbidity^1,2^. Nevertheless, there is no evidence supporting the use of CS without a clear medical indication^5^. On the other hand, CS without medical indications is associated with the increased risks of maternal death, admission to ICU, blood transfusion and hysterectomy^6^.

As a major surgical procedure, CS not only predisposes short term adverse events to pregnant women, i.e. higher rates of haemorrhage, transfusions received, infections, prolonged hospital stays and in infants i.e. higher rates of infection, respiratory complications and admission to neonatal intensive care, but also long-term obstetric risks in the subsequent pregnancy such as placenta previa, morbidly adherent placenta, and uterine rupture^3–8^. The risks of adverse outcomes following CS increase with an increased number of CS^9^.

Over the past decade, the CS rates have continued to trend upward^10^. The major contributions to the high CS rate are from previous CS and CS that were performed in nulliparous women^11–15^. A previous CS makes the greatest contribution to the overall rate of cesarean with a relative contribution ranging from 15.4% to 67.7%^11–15^. A relative contribution of CS performed among nulliparous women to the overall CS rate varies from 17.2% to 41.6%^11–15^. The notably high CS rates among nulliparous women may be associated with increased use of CS without medical indication and inappropriate induction of labour^16^. Based on these findings, the number of deliveries after previous CS therefore is on the rise that constitutes a growing concern over the potential adverse pregnancy outcomes among women with a prior history of CS. This study was conducted to determine the association of a previous CS on the adverse maternal and neonatal outcomes of subsequent pregnancy.

## Results

### Characteristics of study population

**Figure 1:**
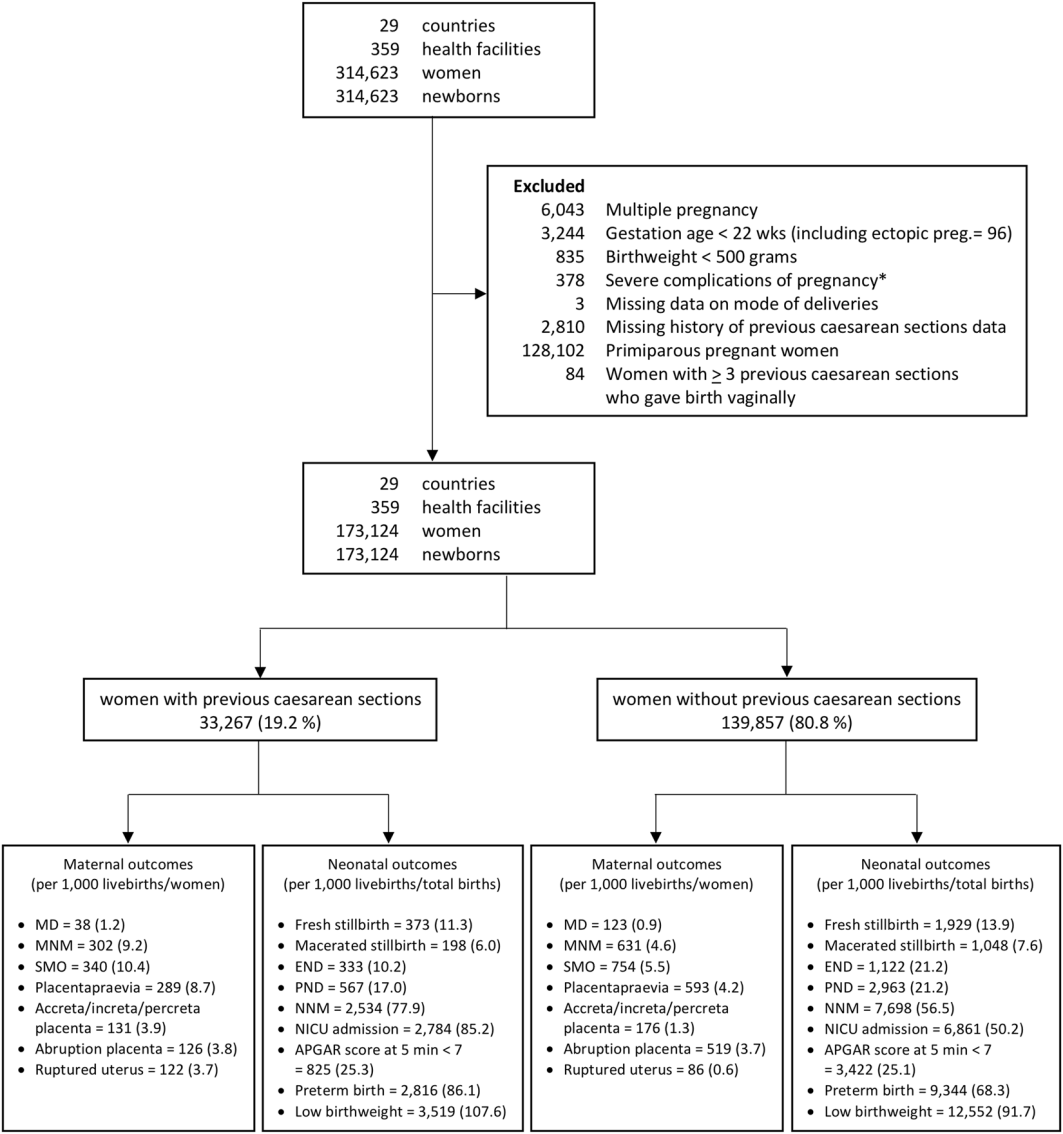
Population flow chart. Note: *admission for near miss/maternal death who did not experience a delivery or abortion at participating facilities. MNM = maternal near miss, SMO = severe maternal outcome, NNM = neonatal near miss, NICU = neonatal intensive care unit.

Of the 173,124 multiparous pregnant women included in this analysis, 33,267 (19.2%) had a history of previous CS (Fig. 1). Table 1 displays the number of previous CS among pregnant women included in this study stratified by countries. Of the 33,267 multiparous pregnant women with previous CS, 7,306 (22.0%) had undergone more than one previous CS. Prevalence of previous CS was highest in Paraguay (37.5%), Brazil (36.5%), Mexico (32.6%), Lebanon (31.9%), and Peru (31.5%) while the lowest was noted in Afghanistan (3.7%), Niger (5.3%), Nicaragua (7.3%), Cambodia (7.4%), and Angola (8.2%).

**Table 1:** Number and percentages of multiparous women with previous caesarean sections by countries.

Countries	No. of Previous caesarean section														Total	CS (%)
	0	(%)	1	(%)	2	(%)	3	(%)	4	(%)	5	(%)	>5	(%)		
PRY	1,269	(0.9)	530	(2.0)	173	(2.9)	51	(4.8)	7	(3.8)	2	(5.7)	0	(0.0)	2,032	(37.5)
BRA	2,238	(1.6)	1,009	(3.9)	217	(3.6)	50	(4.7)	10	(5.5)	2	(5.7)	0	(0.0)	3,526	(36.5)
MEX	4,465	(3.2)	1,730	(6.7)	384	(6.4)	36	(3.4)	5	(2.7)	0	(0.0)	0	(0.0)	6,620	(32.6)
LBN	1,722	(1.2)	501	(1.9)	205	(3.4)	72	(6.7)	23	(12.6)	4	(11.4)	1	(12.5)	2,528	(31.9)
PER	5,884	(4.2)	2,092	(8.1)	544	(9.0)	73	(6.8)	3	(1.6)	0	(0.0)	0	(0.0)	8,596	(31.5)
PAK	5,916	(4.2)	1,666	(6.4)	661	(11.0)	201	(18.8)	28	(15.4)	3	(8.6)	0	(0.0)	8,475	(30.2)
VNM	5,115	(3.7)	2,045	(7.9)	160	(2.7)	6	(0.6)	0	(0.0)	0	(0.0)	0	(0.0)	7,326	(30.2)
JOR	673	(0.5)	147	(0.6)	55	(0.9)	25	(2.3)	16	(8.8)	4	(11.4)	1	(12.5)	921	(26.9)
LKA	6,995	(5.0)	2,072	(8.0)	485	(8.1)	9	(0.8)	0	(0.0)	0	(0.0)	0	(0.0)	9,561	(26.8)
ECU	3,867	(2.8)	903	(3.5)	402	(6.7)	72	(6.7)	11	(6.0)	2	(5.7)	1	(12.5)	5,258	(26.5)
CHN	3,662	(2.6)	1,209	(4.7)	36	(0.6)	0	(0.0)	0	(0.0)	0	(0.0)	0	(0.0)	4,907	(25.4)
QAT	2,206	(1.6)	510	(2.0)	154	(2.6)	53	(5.0)	12	(6.6)	7	(20.0)	2	(25.0)	2,944	(25.1)
OPT	527	(0.4)	104	(0.4)	36	(0.6)	20	(1.9)	8	(4.4)	3	(8.6)	0	(0.0)	698	(24.5)
THA	3,463	(2.5)	899	(3.5)	115	(1.9)	6	(0.6)	0	(0.0)	0	(0.0)	0	(0.0)	4,483	(22.8)
ARG	3,507	(2.5)	764	(2.9)	160	(2.7)	61	(5.7)	18	(9.9)	2	(5.7)	2	(25.0)	4,514	(22.3)
KEN	9,174	(6.6)	1,730	(6.7)	413	(6.9)	69	(6.5)	2	(1.1)	0	(0.0)	0	(0.0)	11,388	(19.4)
PHL	4,892	(3.5)	831	(3.2)	279	(4.6)	21	(2.0)	2	(1.1)	0	(0.0)	0	(0.0)	6,025	(18.8)
MNG	3,223	(2.3)	561	(2.2)	135	(2.2)	6	(0.6)	0	(0.0)	0	(0.0)	0	(0.0)	3,925	(17.9)
COD	4,730	(3.4)	689	(2.7)	237	(3.9)	84	(7.9)	16	(8.8)	2	(5.7)	0	(0.0)	5,758	(17.9)
JPN	1,289	(0.9)	227	(0.9)	40	(0.7)	1	(0.1)	1	(0.5)	0	(0.0)	0	(0.0)	1,558	(17.3)
IND	13,114	(9.4)	2,299	(8.9)	378	(6.3)	14	(1.3)	1	(0.5)	0	(0.0)	0	(0.0)	15,806	(17.0)
UGA	4,598	(3.3)	553	(2.1)	153	(2.5)	66	(6.2)	9	(4.9)	2	(5.7)	0	(0.0)	5,381	(14.6)
NPL	4,158	(3.0)	552	(2.1)	51	(0.8)	0	(0.0)	0	(0.0)	0	(0.0)	0	(0.0)	4,761	(12.7)
NGA	6,765	(4.8)	686	(2.6)	210	(3.5)	30	(2.8)	4	(2.2)	0	(0.0)	0	(0.0)	7,695	(12.1)
AGO	6,140	(4.4)	448	(1.7)	91	(1.5)	8	(0.7)	2	(1.1)	1	(2.9)	0	(0.0)	6,690	(8.2)
KHM	1,885	(1.3)	138	(0.5)	12	(0.2)	0	(0.0)	0	(0.0)	0	(0.0)	0	(0.0)	2,035	(7.4)
NIC	2,845	(2.0)	202	(0.8)	22	(0.4)	0	(0.0)	0	(0.0)	0	(0.0)	0	(0.0)	3,069	(7.3)
NER	7,737	(5.5)	341	(1.3)	74	(1.2)	10	(0.9)	3	(1.6)	1	(2.9)	1	(12.5)	8,167	(5.3)
AFG	17,798	(12.7)	523	(2.0)	130	(2.2)	25	(2.3)	1	(0.5)	0	(0.0)	0	(0.0)	18,477	(3.7)
Total	139,857	(100.0)	25,961	(100.0)	6,012	(100.0)	1,069	(100.0)	182	(100.0)	35	(100.0)	8	(100.0)	173,124	(19.2)

Note: CS = caesarean section; PRY = Paraguay; BRA = Brazil; MEX = Mexico.

LBN = Lebanon; PER = Peru; PAK = Pakistan; VNM = VietNam; JOR = Jordan; LKA = Sri Lanka; ECU = Ecuador; CHN = China; QAT = Qatar; OPT = Palestinia; THA = Thailand; ARG = Argentina; KEN = Kenya; PHL = Philippines; MNG = Mongolia; COD = of the Congo; JPN = Japan; IND = India; UGA = Uganda; NPL = Nepal; NGA = Nigeria; AGO = Angola; KHM = Cambodia; NIC = Nicaragua; NER = Niger; AFG = Afghanistan.

During the study period, 45,232 (26.1%) women underwent CS. Of the 33,267 women with previous CS, 26,930 (80.9%) underwent repeated CS (Table 2). Table 3 presents the maternal and neonatal characteristics, medical comorbidities, and obstetric complications of all women and cross-tabulated with the history of previous CS.

**Table 2:** Index mode of delivery of multiparous women stratified by previous history of caesarean section.

Index pregnancy mode of delivery	No. of Previous caesarean section						Total
	0	(%)	1	(%)	2	(%)	
Vaginal delivery	121,555	(86.9)	6,006	(23.1)	331	(4.5)	127,892
Caesarean section	18,302	(13.1)	19,955	(76.9)	6,975	(95.5)	45,232
Total	139,857	(100.0)	25,961	(100.0)	6,016	(100.0)	173,124

**Table 3:** Maternal and neonatal characteristics of multiparous women with and without previous caesarean sections.

Baseline characteristics	All women (n = 173,124)		Women without previous CS (n = 139,857)		Women with previous CS (n = 33,267)	
	n	(%)	n	(%)	n	(%)
**Maternal characteristics**						
Age (years); available	172,557		139,352		33,205	
<20	4,495	(2.6)	3,798	(2.7)	697	(2.1)
20–34	136,860	(79.3)	110,708	(79.4)	26,152	(78.8)
≥35	31,202	(18.1)	24,846	(17.8)	6,356	(19.1)
Marital status; available	171,836		138,814		33,022	
With partner	159,427	(92.8)	128,624	(92.7)	30,803	(93.3)
Without partner	12,409	(7.2)	10,190	(7.3)	2,219	(6.7)
Years attend school; available	159,926		128,871		31,055	
0	32,743	(20.5)	29,806	(23.1)	2,937	(9.5)
1–6	25,609	(16.0)	21,477	(16.7)	4,132	(13.3)
>6	101,574	(63.5)	77,588	(60.2)	23,986	(77.2)
Parity; available	172,964		139,774		33,190	
1–2	125,239	(72.4)	97,595	(69.8)	27,644	(83.3)
>2	47,725	(27.6)	42,179	(30.2)	5,546	(16.7)
Onset of labour; available	172,884		139,720		33,164	
Spontaneous labour	136,372	(78.9)	119,393	(85.5)	16,979	(51.2)
Induced labour	15,130	(8.8)	13,464	(9.6)	1,666	(5.0)
No labour	21,382	(12.4)	6,863	(4.9)	14,519	(43.8)
Fetal presentation; available	172,837		139,668		33,169	
Cephalic	165,595	(95.8)	134,477	(96.3)	31,118	93.8)
Breech	5,528	(3.2)	4,051	(2.9)	1,477	4.5)
Others	1,714	(1.0)	1,140	(0.8)	574	1.7)
Medical condition; available	173,124		139,857		33,267	
Anaemia	2506	(1.4)	1735	(1.2)	771	(2.3)
HIV	833	(0.5)	664	(0.5)	169	(0.5)
Hypertension	805	(0.5)	553	(0.4)	252	(0.8)
Others	1047	(0.6)	752	(0.5)	295	(0.9)
Obstetric complication; available	173,124		139,857		33,267	
Pre-eclampsia/Eclampsia	3506	(2.0)	2570	(1.8)	936	(2.8)
**Neonatal characteristics**						
Births; available	173,068		139,807		33,261	
Livebirths	169,520	(98.0)	136,830	(97.9)	32,690	(98.3)
Stillbirths	3,548	(2.1)	2,977	(2.1)	571	(1.7)
Gestational age (weeks); available	173,124		139,857		33,267	
Preterm (<37)	12,160	(7.0)	9,344	(6.7)	2,816	(8.5)
Postterm (≥42)	2,756	(1.6)	2,378	(1.7)	378	(1.1)

Note: ^*^Including Malaria/Dengue, Heart, Lung, Renal and Hepatic disease. CS = caesarean section, HIV = human immunodeficiency virus.

Almost all facilities (90%) had blood bank service. An intensive care unit was available in approximately 56% of participating facilities. The majority of countries of participating facilities (51.7%) were reported to have a moderate maternal mortality ratio (MMR). Approximately 17.2% had high and 24.1% very high MMRs of countries of participating facilities^17^.

### Association between previous CS and maternal outcomes

Table 4 presents the adverse maternal outcomes in this study. Overall, the common maternal adverse events were severe maternal outcome (SMO) of 6.5 per 1,000 livebirths, maternal near miss (MNM) of 5.5 per 1,000 livebirths, and placenta previa of 5.1 per 1,000 women. There were 161 maternal deaths, accounting for an overall ratio of 0.9 per 1,000 livebirths.

**Table 4:** Pregnancy outcomes stratified by previous caesarean section.

Outcomes	All women (n = 173,124)			Women without previous CS (n = 139,857)			Women with previous CS (n = 33,267)		
	Event	Total/Livebirths	Rate/Ratio	Event	Total/Livebirths	Rate/Ratio	Event	Total/Livebirths	Rate/Ratio
**Maternal outcomes**									
Maternal death	161	169,520	(0.9)^*^	123	136,830	(0.9)^*^	38	32,690	(1.2)^*^
Maternal near miss	933	169,520	(5.5)^*^	631	136,830	(4.6)^*^	302	32,690	(9.2)^*^
Severe maternal outcome	1,094	169,520	(6.5)^*^	754	136,830	(5.5)^*^	340	32,690	(10.4)^*^
Placenta praevia	883	173,124	(5.1)^**^	593	139,857	(4.2)^**^	289	33,267	(8.7)^**^
Accreta/increta/percreta placenta	307	173,124	(1.8)^**^	176	139,857	(1.3)^**^	131	33,267	(3.9)^**^
Abruption placenta	645	173,124	(3.7)^**^	519	139,857	(3.7)^**^	126	33,267	(3.8)^**^
Ruptured uterus	208	173,124	(1.2)^**^	86	139,857	(0.6)^**^	122	33,267	(3.7)^**^
**Neonatal outcomes**									
Fresh stillbirth	2,302	171,822	(13.4)^***^	1,929	138,759	(13.9)^***^	373	33,063	(11.3)^***^
Macerated stillbirth	1,246	170,766	(7.3)^***^	1,048	137,878	(7.6)^***^	198	32,888	(6.0)^***^
Early neonatal death	1,455	169,388	(8.6)^*^	1,122	136,718	(8.2)^*^	333	32,670	(10.2)^*^
Perinatal death	3,530	173,103	(20.4)^***^	2,963	139,837	(21.2)^***^	567	33,266	(17.0)^*^
Neonatal near miss	10,232	168,676	(60.7)^*^	7,698	136,146	(56.5)^*^	2,534	32,530	(77.9)^*^
NICU admission	9,645	169,438	(56.9)^*^	6,861	136,759	(50.2)^*^	2,784	32,679	(85.2)^*^
Apgar score at 5 min <7	4,247	168,889	(25.1)^*^	3,422	136,317	(25.1)^*^	825	32,572	(25.3)^*^
Preterm birth (<37 weeks)	12,160	169,520	(71.7)^*^	9,344	136,830	(68.3)^*^	2,816	32,690	(86.1)^*^
Low birthweight (<2500 g)	16,071	169,520	(94.8)^*^	12,552	136,830	(91.7)^*^	3,519	32,690	(107.6)^*^

Note: ^*^Per 1,000 livebirths. ^**^Per 1,000 women. ^***^Per 1,000 total births. CS = caesarean section, NICU = neonatal intensive care unit.

As compared with those without history of previous CS, pregnant women with a previous CS were significantly associated with increased risks of uterine rupture (aOR 7.74; 95% CI 5.48, 10.92); morbidly adherent placenta (aOR 2.60; 95% CI 1.98, 3.40), MNM (aOR 1.91; 95% CI 1.59, 2.28), SMO (aOR 1.80; 95% CI 1.52, 2.13), and placenta previa (aOR 1.76; 95% CI 1.49, 2.07). There were no significant differences between the two comparison groups in terms of maternal death and placental abruption (Table 5).

**Table 5:** Associations between previous caesarean sections and pregnancy outcomes among multiparous pregnant women.

Outcomes	crude OR	(95% CI)	p-value	aOR ^*^	(95% CI)	p-value
**Maternal outcomes**						
Maternal death	1.29	(0.87, 1.92)	0.203	1.10	(0.70, 1.72)	0.682
Maternal near miss	1.90	(1.62, 2.22)	<0.001	1.91	(1.59, 2.28)	<0.001
Severe maternal outcome	1.81	(1.57, 2.09)	<0.001	1.80	(1.52, 2.13)	<0.001
Placenta praevia	1.82	(1.56, 2.12)	<0.001	1.76	(1.49, 2.07)	<0.001
Accreta/increta/percreta placenta	2.85	(2.23, 3.65)	<0.001	2.60	(1.98, 3.40)	<0.001
Abruption placenta	1.01	(0.81, 1.26)	0.922	0.99	(0.78, 1.25)	0.939
Ruptured uterus	6.89	(5.02, 9.47)	<0.001	7.74	(5.48, 10.92)	<0.001
**Neonatal outcomes**						
Fresh stillbirth	0.98	(0.86, 1.11)	0.710	0.95	(0.83, 1.09)	0.488
Macerated stillbirth	0.78	(0.66, 0.92)	0.003	0.80	(0.67, 0.95)	0.012
Early neonatal death	1.12	(0.98, 1.28)	0.090	1.08	(0.94, 1.25)	0.295
Perinatal death	0.90	(0.82, 0.99)	0.043	0.90	(0.81, 1.01)	0.070
Neonatal near miss	1.24	(1.17, 1.30)	<0.001	1.19	(1.12, 1.26)	<0.001
NICU admission	1.33	(1.26, 1.40)	<0.001	1.31	(1.23, 1.39)	<0.001
Apgar score at 5 min <7	1.13	(1.03, 1.24)	0.011	1.06	(0.95, 1.17)	0.289
Preterm birth (<37 weeks)	1.08	(1.02, 1.13)	0.003	1.07	(1.01, 1.14)	0.028
Low birthweight (<2500 g)^**^	1.04	(0.99, 1.08)	0.105	1.00	(0.96, 1.05)	0.885

Multiparous women without previous caesarean section is the reference. Note: ^*^Adjusted for individual level: maternal age, marital status, years of maternal school attendance, parity, indirect causes (anaemia, malaria/dengue, HIV, heart disease, lung disease, renal disease, hepatic disease, and chronic hypertension), pre-eclampsia/eclampsia, fetal presentation, birthweight; facility level: blood bank, adult intensive care unit, neonatal intensive care unit. ^**^Excluded birthweight from the model.

CI = confidence interval, NICU = neonatal intensive care unit, aOR = adjusted OR.

### Association between previous CS and neonatal outcomes

The most common adverse neonatal outcome was low birth weight (94.8 per 1,000 livebirths), followed by preterm birth (71.7 per 1,000 livebirths), neonatal near miss (NNM 60.7 per 1,000 livebirths), and neonatal intensive care unit (NICU) admission (56.9 per 1,000 livebirths). The overall rates of early neonatal and perinatal deaths were 8.6 per 1,000 livebirths and 20.4 per 1,000 total births (Table 4). Newborns of women with previous CS were significantly associated with increased risk of NICU admission (aOR 1.31; 95% CI 1.23, 1.39), neonatal near miss (aOR 1.19; 95% CI 1.12, 1.26), preterm birth (aOR 1.07; 95% CI 1.01, 1.14), and decreased risk of macerated stillbirth (aOR 0.80; 95% CI 0.67, 0.95). There were no significantly associated increased risks in women with a previous CS of fresh stillbirths, END, perinatal death, low Apgar score, and low birthweight (Table 5).

### Association between number of previous CS and pregnancy outcomes

Table 6 presents association between number of previous CS and adverse pregnancy outcomes. The risks of MNM, SMO, placenta praevia, and morbidly adherent placenta in subsequent pregnancy increased markedly with the increasing number of previous CS. There were no marked differences in the magnitude of association between the risk of adverse neonatal outcomes and number of previous CS.

**Table 6:** Association between number of previous caesarean sections and pregnancy outcomes among multiparous pregnant women.

Pregnancy outcomes	Crude analysis				Adjusted analysis^*^			
	1 CS		2 CS		1 CS		2 CS	
	OR (95% CI)	p-value	OR (95% CI)	p-value	OR (95% CI)	p-value	OR (95% CI)	p-value
**Maternal outcomes**								
Maternal death	1.01 (0.63, 1.62)	0.975	2.28 (1.29, 4.05)	0.005	0.92 (0.54, 1.58)	0.764	1.59 (0.81, 3.09)	0.174
Maternal near miss	1.58 (1.32, 1.89)	<0.001	3.06 (2.43, 3.84)	<0.001	1.62 (1.32, 1.99)	<0.001	2.75 (2.11, 3.57)	<0.001
Severe maternal outcome	1.50 (1.27, 1.77)	<0.001	2.94 (2.38, 3.64)	<0.001	1.52 (1.25, 1.85)	<0.001	2.63 (2.05, 3.37)	<0.001
Placenta Praevia	1.47 (1.23, 1.76)	<0.001	3.13 (2.50, 3.91)	<0.001	1.50 (1.23, 1.81)	<0.001	2.51 (1.97, 3.20)	<0.001
Accreta/increta/Percreta placenta	2.09 (1.57, 2.78)	<0.001	6.99 (4.93, 9.92)	<0.001	1.90 (1.39, 2.59)	<0.001	5.85 (3.99, 8.58)	<0.001
Abruption placenta	1.07 (0.84, 1.35)	0.592	0.83 (0.54, 1.28)	0.397	1.15 (0.89, 1.48)	0.283	0.61 (0.38, 0.98)	0.042
Ruptured Uterus	6.79 (4.87, 9.48)	<0.001	7.25 (4.56, 11.51)	<0.001	8.23 (5.71, 11.84)	<0.001	6.50 (3.92, 10.76)	<0.001
**Neonatal outcomes**								
Fresh stillbirth	1.01 (0.88, 1.16)	0.908	0.87 (0.67, 1.12)	0.274	1.05 (0.90, 1.21)	0.555	0.70 (0.54, 0.92)	0.010
Macerated stillbirth	0.74 (0.61, 0.89)	0.001	0.92 (0.69, 1.23)	0.581	0.81 (0.67, 0.99)	0.044	0.75 (0.55, 1.03)	0.072
Early neonatal death	1.03 (0.88, 1.19)	0.743	1.44 (1.16, 1.79)	<0.001	1.02 (0.87, 1.20)	0.805	1.25 (0.98, 1.57)	0.067
Perinatal death	0.90 (0.81, 1.01)	0.066	0.90 (0.75, 1.09)	0.285	0.96 (0.85, 1.09)	0.562	0.74 (0.60, 0.91)	0.004
Neonatal near miss	1.17 (1.10, 1.24)	<0.001	1.47 (1.34, 1.61)	<0.001	1.15 (1.07, 1.23)	<0.001	1.33 (1.20, 1.47)	<0.001
NICU admission	1.25 (1.18, 1.32)	<0.001	1.64 (1.51, 1.80)	<0.001	1.24 (1.16, 1.33)	<0.001	1.53 (1.38, 1.69)	<0.001
Apgar score at 5 min < 7	1.09 (0.98, 1.21)	0.096	1.26 (1.07, 1.48)	0.006	1.04 (0.93, 1.16)	0.523	1.11 (0.93, 1.32)	0.239
Preterm birth (<37 weeks)	0.96 (0.91, 1.02)	0.175	1.49 (1.38, 1.62)	<0.001	0.97 (0.90, 1.03)	0.314	1.42 (1.29, 1.58)	<0.001
Low birthweight (<2500 g)^**^	0.98 (0.93, 1.03)	0.350	1.27 (1.17, 1.37)	<0.001	0.95 (0.90, 0.99)	0.049	1.21 (1.11, 1.31)	<0.001

Multiparous women without previous caesarean section is the reference. Note: ^*^Adjusted for individual level: maternal age, marital status, years of maternal school attendance, indirect causes (anaemia, malaria/dengue, HIV, heart disease, lung disease, renal disease, hepatic disease, and chronic hypertension), pre-eclampsia/eclampsia, fetal presentation, birthweight; facility level: blood bank, adult intensive care unit, neonatal intensive care unit. ^**^Excluded birthweight from the model.

CI = confidence interval, NICU = neonatal intensive care unit, aOR = adjusted OR.

## Discussion

Approximately 19.2% of the study population reported a history of previous CS. Rates of previous CS, however, varied widely ranging from only 3.7% in Afghanistan to 37.5% in Paraguay. This reflects a wide variation of previous CS rates across the participating countries. After controlling the potential confounders at both individual and facility levels, it was observed that there were significant association of various adverse pregnancy outcomes among pregnant women with a previous CS. A previous CS is significantly associated with increased risks of uterine rupture, morbidly adherent placenta, MNM, SMO, and placenta previa. There was no significant difference, however, in rate of maternal death between pregnant women with a previous CS and those who had none. For neonatal outcomes, a previous CS was significantly associated with increased risks of NICU admission, NNM, preterm birth, and decreased risk of macerated stillbirth. There were no significantly increased risks of, fresh stillbirth, early neonatal death, perinatal death, low Apgar score, and low birthweight between the two comparison groups.

In the present analysis, uterine rupture was the outcome with the strongest association with a previous CS. The risk of uterine rupture was nearly 7 times higher for women with previous CS when compared to those without previous CS. Uterine rupture during pregnancy and delivery is amongst the most devastating obstetric complications as it frequently results in life-threatening maternal and foetal compromises. Previous secondary analysis of the WHOMCS dataset reported that uterine rupture was significantly associated with increases in MNM (aOR, 45.25; 95% CI, 26.45 to 77.42), maternal mortality (aOR, 4.45; 95% CI, 1.15 to 17.26), SMO (aOR, 40.22; 95% CI, 24.01 to 67.36), fresh stillbirth (aOR, 59.56; 95% CI, 38.29 to 92.64), intra-hospital early neonatal death (aOR, 8.95; 95% CI, 3.72 to 21.52) and perinatal death (aOR, 33.34; 95% CI, 21.59 to 51.51)^7^.

The present study observed a significant association between previous CS and the risk of MNM and SMO. MNM and SMO cases are 1.9 times and 1.8 times more frequent in women with previous CS. There was no significant difference, however, between the two comparison groups in risk of maternal death which might be secondary to a remarkably low rate of maternal death in the WHOMCS data. Women being survivors of a life-threatening condition that is a maternal near-miss case, share several clinico-pathologic factors in common with those who died. The WHO near-miss approach recommends incorporating the near-miss cases into enquiries of maternal deaths to better assess the quality of care and its associated factors^18^.

In line with literature, this study showed association of abnormal placentation among women with previous CS^8,19,20^. Previous CS was associated with increased risks of morbidly adherent placenta (2.6 times higher) and placenta previa (1.8 times higher) compared to those without previous CS. This finding is clinically important. A previous cohort study in UK reported that morbidly adherent placenta constituted approximately 40% of the causes leading to peripartum hysterectomy^21^. In addition, 20% of postpartum haemorrhage cases requiring massive blood transfusion were secondary to placenta^22^ praevia.

A previous study reported that infants born to mothers with a previous CS were significantly more likely to require respiratory support and have prolonged hospitalisation for more than 7 days when compared to those infants born to mothers with prior vaginal delivery. There was no difference, however, in neonatal mortality rate between the groups^23^. In the present study, previous CS was associated with increased risk of NICU admission and marginally increased risk of NNM.

Interestingly, this analysis observed a statistically significant association between previous CS and preterm birth. The pragmatic nature of a cross-sectional study *per se* makes this analysis unable to determine the causal relationships between previous CS and this neonatal outcome. Previous study undertaken in a large cohort of pregnant women in the United States also noted a small, but statistically significant association between previous CS and preterm birth. Pregnant women with previous CS were 14% more likely to experience a preterm birth in the second pregnancy (95% CI, 1.12 to 1.16)^24^. These findings highlight that efforts to limit unnecessary CS may lead to lowering the preterm birth rate.

This study analysed the WHOMCS dataset that used pretested, standardized data collection forms collected by well-trained research assistants in institutes with experience from the previous WHO Global Survey to strengthen the quality of data. In term of available knowledge, this study is the most recent and largest multi-country survey data analyzed to determine the impact of previous CS on the risks of key adverse maternal and perinatal outcomes in subsequent pregnancies. The large sample size in this survey yields a notably high precision of summary measures. In addition, this dataset offered outcome measures as per the most recent approach recommended by WHO.

Nonetheless, some limitations of this study are worthy of note. First, although data were extracted by intensively trained research assistants using standardized recorded forms, applying medical records as the primary data source, means that the possibility of missing data and/or errors in these records cannot be discarded. Secondly, the WHOMCS was conducted in 359 health facilities from 29 countries in Africa, Asia, Latin America, and the Middle East. All of these countries except Japan are in low or low-middle income countries (LMICs). In addition, the WHOMCS data were obtained primarily from secondary and tertiary health facilities which were likely to have an over-representation of complicated pregnancies. Extrapolation of these findings to facilities of different backgrounds, i.e. high income countries or primary health facilities, may be limited. Thirdly, this study attempted an adjustment for potential confounders to demonstrate a possible independent association of previous CS on adverse pregnancy outcomes. However, information on some other variables that might be associated with pregnancy outcomes, including adequacy of antenatal care, indications of previous CS, nutritional status, smoking, obesity and diabetes were not available. This was because this WHOMCS focused on the assessment of maternal death and maternal near miss. Data collection on potential confounding factors was therefore not very comprehensive. The absence of data on indication of previous CS including whether the CS was medically indicated, or whether the indication for the original CS still exists for subsequent pregnancies. This limitation should be cautiously considered in the interpretation of findings.

Based on the heightened risks of MNM and SMO, women with a history of previous CS should be regarded as a group of high-risk pregnancies requiring a specialised care bundle from multidisciplinary trained providers. Similarly, the greater risks of neonatal near miss and NICU admission associated with previous CS women indicates the need of high quality of cares including an availability of a NICU to minimize long-term neonatal consequences. Primary CS should be performed only when medically necessary. Future researches to delineate an effective strategy for curbing rising CS rates are mandatory to avoid unfavourable pregnancy outcomes that are associated with a previous CS.

The present study did suggest some general trends in that a previous CS is associated with various adverse maternal and neonatal outcomes. These findings however should be cautiously interpreted due to lacking data on indication of previous CS.

## Methods

### Study design and setting

The design of the WHOMCS was described in detail elsewhere^25^. Briefly, WHOMCS was a facility-based survey conducted to determine pregnancy outcomes among health facilities in Africa, Asia, Latin America, and the Middle East from May 1, 2010 to December 31, 2011. WHOMCS used a stratified, multistage cluster sampling approach to obtain 359 health facilities in 29 countries. Health facilities were only recruited if they dealt with at least 1000 deliveries per year and had the capacity to provide caesarean section.

Data collection took place at two levels including the individual and facility levels. At the individual level, data of management and the pregnancy outcomes for the women were included in the study, and their respective newborn records, were retrieved from the medical records of the participating facilities by trained data collectors. Data were recorded in the pre-established form at hospital discharge, transfer, or death. Data collectors did not contact the women included in the study; however, data clarification was occasionally required from facility staff. At the facility level, data of characteristics of each health facility, including infrastructure, obstetric and intensive care services, availability of blood bank service, and their ability to treat severe complications, were collected through a specific survey among the healthcare professionals responsible for the participating facilities. Data were collected from the time of admission to death, discharge or 7 days postpartum (whichever came first).

The duration of data collection depended on the number of deliveries per annum of each facility. To reduce variation in cluster size, data were collected for 2 months if they had 6000 deliveries or more per year, and for 3 months if the health facility had fewer than 6000 deliveries per year. If the anticipated sample size for a country was fewer than 3000 women, the data collection period was 4 months in all health facilities.

Online data entries were carried out in each country, either at the health facility or at a central level, depending on the logistics and available infrastructure using the web-based data management system developed by the Centro Rosarino de Estudios Perinatales (CREP), Rosario, Argentina. Monitoring of data validity and consistency were carried out by Data Managers from CREP and Thailand. This study was approved by the WHO Ethics Review Committee and relevant ethics clearance bodies in participating countries.

### Study population

The study population was all consecutive multiparous pregnant women who gave birth to singleton babies, either live births or stillbirths, at the 359 participating facilities. Figure 1 displays the population flow chart of subjects and exclusion criteria in the present analysis. We additionally excluded women with at least three previous CS who gave birth vaginally as this is unusual practice at present.

### Variables and definitions

Multiparous pregnant women who had a history of previous CS were considered the exposure group. The comparison group was pregnant women without history of previous CS.

Pregnancy adverse outcomes were categorized into maternal and neonatal outcomes. The maternal outcomes were MNM, maternal death, and SMO which is a combination of MNM and maternal death. The neonatal outcomes included stillbirth, early neonatal death, perinatal death, NNM, NICU admission, an Apgar score at 5 min of less than 7, low birthweight (<2500 g), and preterm birth (<37 weeks of gestation).

Potential confounding factors were abstracted from both facility and individual levels. Potential confounding factors at the facility level included the availability of a blood bank, an adult intensive care unit for severe maternal conditions, and a NICU for severe neonatal conditions. Potential confounding factors at an individual level were maternal demographic and labor characteristics. Maternal demographic information included marital status, maternal education in years of school attendance, parity, and indirect causes (aneamia, malaria/dengue, HIV, heart disease, lung disease, renal disease, hepatic disease, and chronic hypertension). Labour characteristics included fetal presentation, and mode of delivery. However, we did not have information about indications of these previous CS.

### Statistical analysis

Frequencies were used to describe country groups, baseline maternal and neonatal characteristics, and maternal and neonatal outcomes. Details of maternal and neonatal outcomes were also stratified by prior history of CS. The association between previous CS and maternal and neonatal outcomes were analyzed using a two-level logistic regression model by package lme4 of R software^26^. This procedure was carried out to account for clustering effects of health facilities and confounding effect at both health facility and individual levels for estimating risks of previous CS for each outcome. Women who never had CS represented the reference group.

Associated risks of previous CS for individual outcomes were presented by adjusted odds ratios (aORs), with corresponding 95% confidence intervals. Adjusted confounders in the models were chosen based on literature review. Statistical analyses were performed using R software.

### Ethical approval

The HRP Specialist Panel on Epidemiological Research reviewed and approved the study protocol for technical content. This study was approved by the World Health Organization Ethical Review Committee and the relevant ethical clearance mechanisms in all countries (protocol ID A65661; 27 October 2009). This study adhered to the principles of the Declaration of Helsinki. Informed consent was formally waived by the WHO Ethical Review committee. Therefore, written consent from individual women was not required as data collectors extracted data from medical records and did not contact the individual women.

## Data Availability

The datasets generated and/or analyzed during the current study are not publicly available due to they belonged to Department of Reproductive Health and Research, The World Health Organization but could be available from WHO on reasonable request.

## References

[1] Molina G (2015). Relationship Between Cesarean Delivery Rate and Maternal and Neonatal Mortality. JAMA.

[2] Thomas S, Meadows J, McQueen KA (2016). Access to Cesarean Section Will Reduce Maternal Mortality in Low-Income Countries: A Mathematical Model. World J Surg.

[3] Shah A (2009). Cesarean delivery outcomes from the WHO global survey on maternal and perinatal health in Africa. Int J Gynaecol Obstet.

[4] Villar J (2006). Caesarean delivery rates and pregnancy outcomes: the 2005 WHO global survey on maternal and perinatal health in Latin America. Lancet.

[5] Lumbiganon P (2010). Method of delivery and pregnancy outcomes in Asia: the WHO global survey on maternal and perinatal health 2007-08. Lancet.

[6] Souza JP (2010). Caesarean section without medical indications is associated with an increased risk of adverse short-term maternal outcomes: the 2004-2008 WHO Global Survey on Maternal and Perinatal Health. BMC Med.

[7] Motomura K (2017). Incidence and outcomes of uterine rupture among women with prior caesarean section: WHO Multicountry Survey on Maternal and Newborn Health. Sci Rep.

[8] Silver RM (2015). Abnormal Placentation: Placenta Previa, Vasa Previa, and Placenta Accreta. Obstet Gynecol.

[9] Marshall NE, Fu R, Guise JM (2011). Impact of multiple cesarean deliveries on maternal morbidity: a systematic review. Am J Obstet Gynecol.

[10] Betran AP (2016). The Increasing Trend in Caesarean Section Rates: Global, Regional and National Estimates: 1990-2014. PLoS One.

[11] Abdel-Aleem H, Shaaban OM, Hassanin AI, Ibraheem AA (2013). Analysis of cesarean delivery at Assiut University Hospital using the Ten Group Classification System. Int J Gynaecol Obstet.

[12] Ferreira EC, Pacagnella RC, Costa ML, Cecatti JG (2015). The Robson ten-group classification system for appraising deliveries at a tertiary referral hospital in Brazil. Int J Gynaecol Obstet.

[13] Tan JK, Tan EL, Kanagalingan D, Tan LK (2015). Rational dissection of a high institutional cesarean section rate: an analysis using the Robson Ten Group Classification System. J Obstet Gynaecol Res.

[14] Triunfo S, Ferrazzani S, Lanzone A, Scambia G (2015). Identification of obstetric targets for reducing cesarean section rate using the Robson Ten Group Classification in a tertiary level hospital. Eur J Obstet Gynecol Reprod Biol.

[15] Kankoon N (2018). Cesarean rates and severe maternal and neonatal outcomes according to the Robson 10-Group Classification System in Khon Kaen Province, Thailand. Int J Gynaecol Obstet.

[16] Vogel JP (2015). Use of the Robson classification to assess caesarean section trends in 21 countries: a secondary analysis of two WHO multicountry surveys. *Lancet*. Glob Health.

[17] World Health Organization. Maternal mortality in 2000: estimates developed by WHO, UNICEF and UNFPA. Available from, http://www.who.int/iris/handle/10665/42930 (2004).

[18] Pattinson R, Say L, Souza JP, Broek N, Rooney C (2009). WHO maternal death and near-miss classifications. Bull World Health Organ.

[19] Getahun D, Oyelese Y, Salihu HM, Ananth CV (2006). Previous cesarean delivery and risks of placenta previa and placental abruption. Obstet Gynecol.

[20] Morlando M (2013). Placenta accreta: incidence and risk factors in an area with a particularly high rate of cesarean section. Acta Obstet Gynecol Scand.

[21] Knight M (2007). Peripartum hysterectomy in the UK: management and outcomes of the associated haemorrhage. BJOG.

[22] Green L (2016). The epidemiology and outcomes of women with postpartum haemorrhage requiring massive transfusion with eight or more units of red cells: a national cross-sectional study. BJOG.

[23] Galyean AM, Lagrew DC, Bush MC, Kurtzman JT (2009). Previous cesarean section and the risk of postpartum maternal complications and adverse neonatal outcomes in future pregnancies. J Perinatol.

[24] Williams CM (2018). Previous cesarean delivery associated with subsequent preterm birth in the United States. Eur J Obstet Gynecol Reprod Biol.

[25] Souza JP, Gulmezoglu AM, Carroli G, Lumbiganon P, Qureshi Z (2011). The world health organization multicountry survey on maternal and newborn health: study protocol. BMC Health Serv Res.

[26] Bates D, Maechler M, Bolker B, Walker SW (2015). Fitting linear mixed-effects models using lme4. J Stat Softw.

